# Supplementary material for: Thermoplasmonic neural chip platform for in situ manipulation of neuronal connections in vitro
Source: Nat Commun. 2020 Dec 9;11:6313. doi: 10.1038/s41467-020-20060-z (PMC7726146; doi:10.1038/s41467-020-20060-z)
Supplement: Supplementary file 2 — Supplementary information [file 41467_2020_20060_MOESM2_ESM.pdf]

[Supplementary Information]

Thermoplasmonic neural chip platform for *in situ* manipulation of neuronal connections *in vitro*

Nari Hong and Yoonkey Nam*

1. Department of Bio and Brain Engineering, Korea Advanced Institute of Science and Technology (KAIST), Daejeon 34141, Republic of Korea
2. KAIST Institute for Health Science and Technology, Korea Advanced Institute of Science and Technology (KAIST), Daejeon 34141, Republic of Korea

*Corresponding address:

291 Daehak-ro, Yuseong-gu,

Daejeon 34141, Republic of Korea.

Tel: +82 42 350 4322

Fax: +82 42 350 4310

ynam@kaist.ac.kr

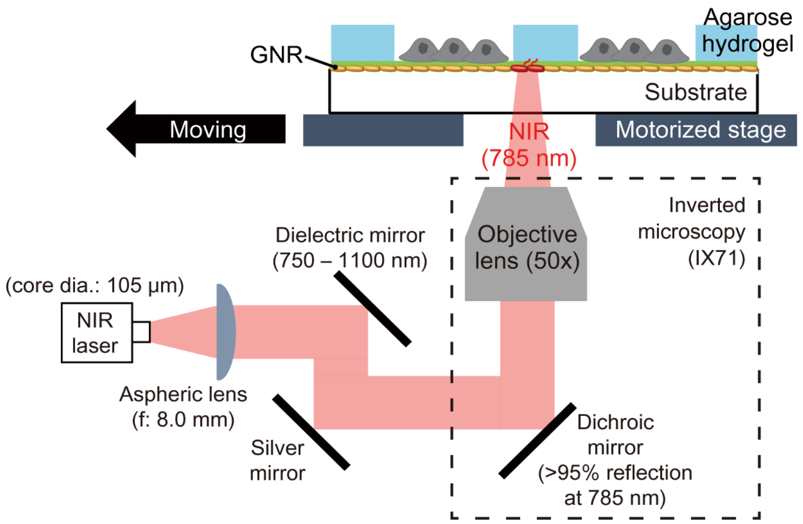

Supplementary Figure 1. Schematics of the NIR illumination system.

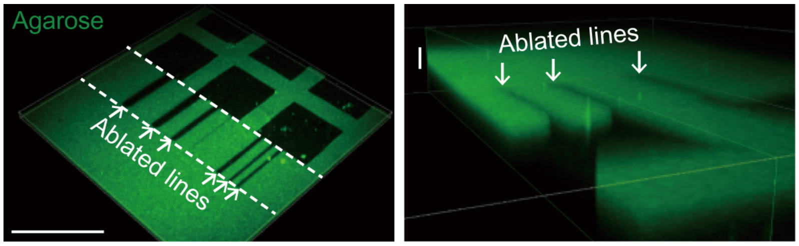

Supplementary Figure 2. Fluorescent images of agarose hydrogel using confocal microscopy. Scale bar: 500 µm (left) and 10 µm (right). The images are representative of two replicates.

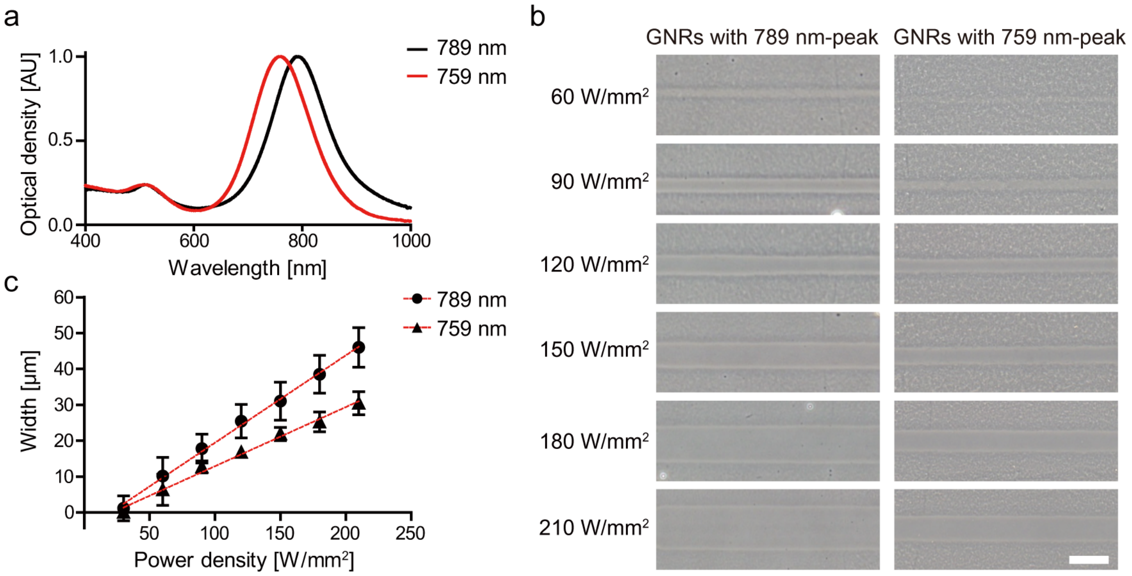

Supplementary Figure 3. Comparison of two different GNRs for the thermoplasmonic ablation of agarose hydrogel. **a** Absorbance spectra of GNRs with a longitudinal peak of 789 nm and 759 nm. **b** Phase-contrast images of ablated regions. Scale bar: 50 µm. **c** Ablated width of agarose hydrogel against power density for two different GNRs. The ablated width when using GNRs with 789 nm-peak was larger than that when using GNRs with 759 nm-peak. n = 9 (789 nm) ablated lines in three independent experiments and 4 (759 nm) ablated lines in two independent experiments. Data are presented as mean ± standard deviation. Source data are provided as a Source Data file.

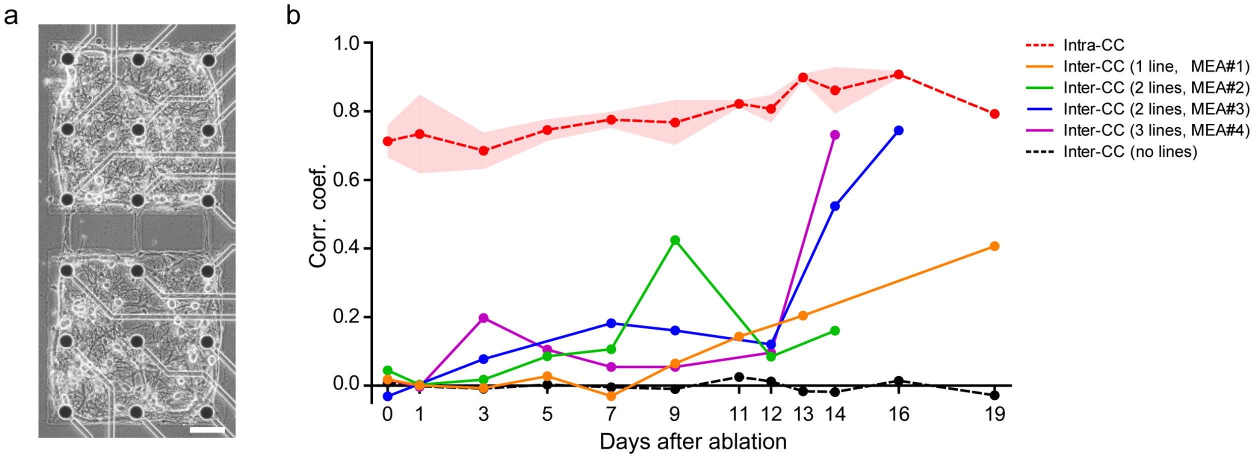

Supplementary Figure 4. Network synchronization in large networks connected through various numbers of interconnection lines. **a** A patterned network with three interconnection lines (MEA#4). Agarose micropatterns were 500 µm by 500 µm with spacing of 100 µm. Scale bar: 100 µm. The images shown are representative of four independent experiments. **b** Correlation coefficient values within (intra-CC) and between (inter-CC) networks. Orange, green, blue, and purple graphs show inter-CC between connected networks with one, two, two, and three-interconnection lines, respectively. Each line represents an individual MEA. Intra-CC line and shaded region denote mean and standard deviation of intra-CCs from 4 MEAs, respectively. Source data are provided as a Source Data file.

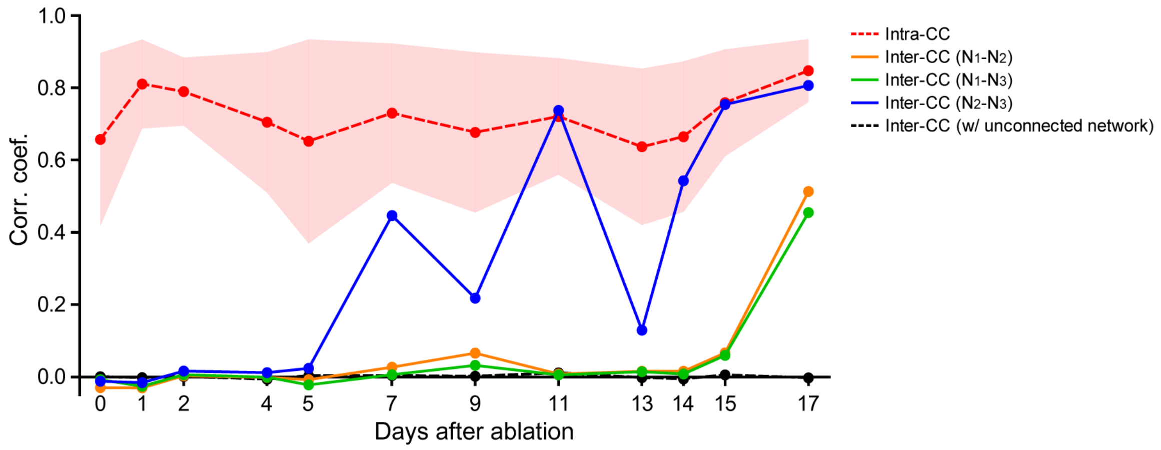

Supplementary Figure 5. Mean correlation coefficient between three networks at different time points after ablation process. Data from Figure 6. The correlation coefficients between N_2 and N_3 (Inter-CC N_2-N_3, blue line) increased from 5 days after the ablation and those of N_1-N_2 (orange line) and N_1-N_3 (green line) rose from 14 days after the ablation. For intra-CCs (red dash line), mean and standard deviation values were denoted by circle symbol and shaded regions. Source data are provided as a Source Data file.

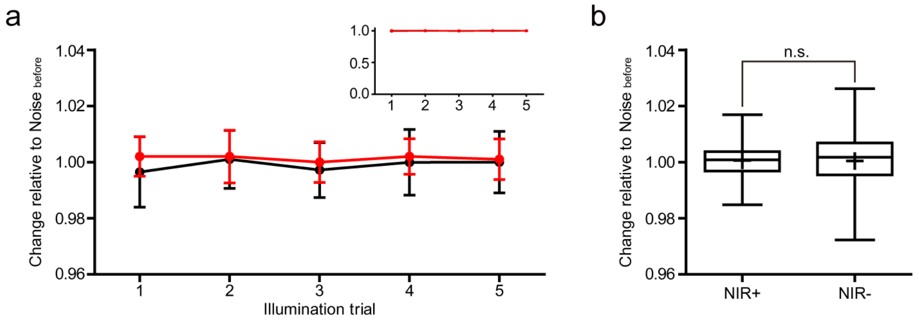

Supplementary Figure 6. Comparison of background noise level after NIR illumination. **a** Change of RMS (root-mean squared) noise values after individual trials normalized by the RMS value before the illumination (243 mW/mm^2). Red and black lines represent the values of electrodes inside (NIR+; n = 18 electrodes) and outside (NIR–; n = 36 electrodes) the NIR-illuminated region, respectively. In case of electrodes in NIR-illuminated region, there was no section showing a significant difference between trials (One-way ANOVA; p = 0.8365). Data are presented as mean ± standard deviation. **b** Change of RMS noise values after five illumination trials relative to those before the illumination. The change of NIR-illuminated region (NIR+) was not significantly different from that of controls (NIR–), implying that the NIR did not cause any significant damage to electrode themselves (Two-sided unpaired t-test; p = 0.9453). n = 18 and 36 electrodes for NIR+ and NIR–, respectively. The mean and median values were shown as '+' and a middle line, respectively. The boxes and whiskers extend from the 25th to 75th percentiles and from min to max values, respectively. Source data are provided as a Source Data file.
