# Supplementary material for: Thermoplasmonic neural chip platform for in situ manipulation of neuronal connections in vitro
Source: Nat Commun. 2020 Dec 9;11:6313. doi: 10.1038/s41467-020-20060-z (PMC7726146; doi:10.1038/s41467-020-20060-z)
Supplement: Supplementary file 3 — Reporting Summary [file 41467_2020_20060_MOESM3_ESM.pdf]

nature research

Corresponding author(s): Yoonkey Nam

Last updated by author(s): Nov 11, 2020

Reporting Summary

Nature Research wishes to improve the reproducibility of the work that we publish. This form provides structure for consistency and transparency in reporting. For further information on Nature Research policies, see our Editorial Policies and the Editorial Policy Checklist.

Statistics

For all statistical analyses, confirm that the following items are present in the figure legend, table legend, main text, or Methods section.

n/a	Confirmed	
☐	☒	The exact sample size (n) for each experimental group/condition, given as a discrete number and unit of measurement
☐	☒	A statement on whether measurements were taken from distinct samples or whether the same sample was measured repeatedly
☐	☒	The statistical test(s) used AND whether they are one- or two-sided<br>*Only common tests should be described solely by name; describe more complex techniques in the Methods section.*
☒	☐	A description of all covariates tested
☒	☐	A description of any assumptions or corrections, such as tests of normality and adjustment for multiple comparisons
☐	☒	A full description of the statistical parameters including central tendency (e.g. means) or other basic estimates (e.g. regression coefficient) AND variation (e.g. standard deviation) or associated estimates of uncertainty (e.g. confidence intervals)
☐	☒	For null hypothesis testing, the test statistic (e.g. F, t, r) with confidence intervals, effect sizes, degrees of freedom and P value noted<br>*Give P values as exact values whenever suitable.*
☒	☐	For Bayesian analysis, information on the choice of priors and Markov chain Monte Carlo settings
☒	☐	For hierarchical and complex designs, identification of the appropriate level for tests and full reporting of outcomes
☐	☒	Estimates of effect sizes (e.g. Cohen's d, Pearson's r), indicating how they were calculated

Our web collection on statistics for biologists contains articles on many of the points above.

Software and code

Policy information about availability of computer code

Data collection	MC_RACK v4.6.2 (Multi Channel Systems MCS GmbH)
Data analysis	MC_RACK v4.6.2 (Multi Channel Systems MCS GmbH), NeuroExplorer v5.112 (Nex Technologies), OfflineSorter v2.8.8 (Plexon Inc.) and MATLAB R2017a (The MathWorks, Inc.) were used for signal analysis. GraphPad Prism v5.03 (GraphPad Software, Inc.) was used for data plotting and statistical analysis.

For manuscripts utilizing custom algorithms or software that are central to the research but not yet described in published literature, software must be made available to editors and reviewers. We strongly encourage code deposition in a community repository (e.g. GitHub). See the Nature Research guidelines for submitting code & software for further information.

Data

Policy information about availability of data

All manuscripts must include a data availability statement. This statement should provide the following information, where applicable:

- Accession codes, unique identifiers, or web links for publicly available datasets
- A list of figures that have associated raw data
- A description of any restrictions on data availability

The data that support the findings of this study are available from the corresponding author upon reasonable request. Source data underlying the figures (Fig. 2b; 3f; 5e, f; 6d, f, i; Supplementary Fig. 3c, 4b, 5, 6) are provided with this paper as a Source Data file.

Field-specific reporting

Please select the one below that is the best fit for your research. If you are not sure, read the appropriate sections before making your selection.

☒ Life sciences ☐ Behavioural & social sciences ☐ Ecological, evolutionary & environmental sciences

For a reference copy of the document with all sections, see nature.com/documents/nr-reporting-summary-flat.pdf

Life sciences study design

All studies must disclose on these points even when the disclosure is negative.

Sample size	No statistical method was used to predetermine the sample size. In consideration of the time required for the experiments and cell viability, the sample sizes were determined to support meaningful conclusions, and they were adequate as the difference between experimental groups was measurable and reproducible. The sample sizes (n) are described in the figure legends.
Data exclusions	No data were excluded.
Replication	All experiments were performed in at least two biological replicates. All attempts were successful.
Randomization	Rats were randomly selected for cell culture. To avoid bias, the tested samples were chosen from different batches.
Blinding	Investigators were not blinded during experiments and outcome assessment. Blinding was not relevant as data acquisition and analysis were automated using the programs.

Reporting for specific materials, systems and methods

We require information from authors about some types of materials, experimental systems and methods used in many studies. Here, indicate whether each material, system or method listed is relevant to your study. If you are not sure if a list item applies to your research, read the appropriate section before selecting a response.

Materials & experimental systems

n/a	Involved in the study	
☐	☒	Antibodies
☒	☐	Eukaryotic cell lines
☒	☐	Palaeontology and archaeology
☐	☒	Animals and other organisms
☒	☐	Human research participants
☒	☐	Clinical data
☒	☐	Dual use research of concern

Methods

n/a	Involved in the study	
☒	☐	ChIP-seq
☒	☐	Flow cytometry
☒	☐	MRI-based neuroimaging

Antibodies

Antibodies used

Primary antibodies:
Anti-MAP2 (1:500, M3696, Sigma-Aldrich), Anti-tau-1 (1:500, MAB3420, Merck Millipore), Anti-beta-III-tubulin (1:500, T2200, Sigma-Aldrich), and Anti-laminin (1:500, L9393, Sigma-Aldrich)

Secondary antibodies (Invitrogen, Thermo Fisher Scientific):
Goat anti-Rabbit IgG, Alexa Fluor 488 (A11008), Goat anti-Rabbit IgG, Alexa Fluor 594 (A11012), Goat anti-Mouse IgG, Alexa Fluor 488 (A11001), and Goat anti-Mouse IgG, Alexa Fluor 594 (A11032)

Validation

Validation is provided on the manufacturer`s website for each product.

- Anti-MAP2 (https://www.sigmaaldrich.com/catalog/product/sigma/m3696)
: Anti-MAP2 antibody is specific for MAP2 in human, mice and rats. Anti-MAP2 antibody is suitable for use in immunohistochemistry

- Anti-tau-1 (https://www.sigmaaldrich.com/catalog/product/mm/mab3420)
: Binds to all known electrophoretic species of tau in human, rat and bovine brain. Anti-Tau-1 Antibody, clone PC1C6 is an antibody against Tau-1 for use in IH & WB with more than 65 product citations.

- Anti-beta-III-tubulin (https://www.sigmaaldrich.com/catalog/product/sigma/t2200)
: Anti-β-Tubulin III recognizes human, mouse,and rat β-tubulin III. Anti-β-Tubulin III antibody produced in rabbit has been used in following studies: Immunofluorescence, Immunostaining, Western blotting.

	- Anti-laminin (https://www.sigmaaldrich.com/catalog/product/sigma/l9393)<br>: The affinity isolated antibody to laminin will react with laminin of human, mammal, avian, reptilian, and amphibian sources. Anti-Laminin antibody has been used in immunohistochemical staining and immunohistochemistry.

Animals and other organisms

Policy information about studies involving animals; ARRIVE guidelines recommended for reporting animal research

Laboratory animals	Sprague-Dawley rats (embryonic day 18) were used in this study.
Wild animals	The study did not involve wild animals.
Field-collected samples	The study did not involve samples collected from the field.
Ethics oversight	All experiments were performed in accordance with the guidance of the Institutional Animal Care and Use Committee (IACUC) of Korea Advanced Institute of Science and Technology (KAIST), and all experimental protocols were approved by IACUC of KAIST.

Note that full information on the approval of the study protocol must also be provided in the manuscript.
